# Application of Wastewater-Based Epidemiology for Tracking Human Exposure to Deoxynivalenol and Enniatins

**DOI:** 10.3390/toxins14020091

**Published:** 2022-01-25

**Authors:** Zane Berzina, Romans Pavlenko, Martins Jansons, Elena Bartkiene, Romans Neilands, Iveta Pugajeva, Vadims Bartkevics

**Affiliations:** 1 Animal Health and Environment “BIOR”, Institute of Food Safety, Lejupes 3, LV-1076 Riga, Latvia; romans.pavlenko@bior.lv (R.P.); martins.jansons@bior.lv (M.J.); iveta.pugajeva@bior.lv (I.P.); vadims.bartkevics@bior.lv (V.B.); 2 Faculty of Chemistry, University of Latvia, Jelgavas 1, LV-1004 Riga, Latvia; 3 Department of Food Safety and Quality, Lithuanian University of Health Sciences, Mickeviciaus 9, LT-44307 Kaunas, Lithuania; elena.bartkiene@lsmuni.lt; 4 Department of Water Engineering and Technology, Riga Technical University, Kalku 1, LV-1658 Riga, Latvia; Romans.Neilands@rtu.lv

**Keywords:** mycotoxins, wastewater, risk assessment, exposure

## Abstract

Wastewater-based epidemiology (WBE) is a promising biomonitoring approach with the potential to provide direct information on human intake and exposure to food contaminants and environmental chemicals. The aim of this study was to apply WBE while employing the normalization method for exploring human exposure to selected mycotoxins according to population biomarker 5-hydroxyindoleacetic acid (5-HIAA). This type of normalization technique has been previously used to detect various other compounds. However, to the best of our knowledge, this is the first study tracking human exposure to mycotoxins. A sensitive analytical methodology was developed to achieve reliable quantification of deoxynivalenol, enniatins, and beauvericin in wastewater (WW) samples. The applicability of the method was evaluated by testing 29 WW samples collected at WW treatment plants in Latvia. With frequency of detection greater than 86%, enniatins B, B1, A, and A1 were revealed in WW samples. The estimated total daily intake for enniatins was in the range of 1.8–27.6 µg/day per person. Free deoxynivalenol (DON) was determined in all analysed WW samples. Based on the average 5-HIAA excretion level and the determined 5-HIAA content in the samples, the intake of DON by the human population of Riga was estimated at 325 ng/kg b.w. day.

## 1. Introduction

The occurrence of mycotoxins in food presents a global problem. 

Although the risks of acute poisoning are low, there is a definite concern about chronic low doses that can lead to cancer and other diseases in the long term [1,2,3]. Thus, maximum acceptable levels have been established for some mycotoxins in different foods by the Commission Regulation 1881/2006 [4] and Health-Based Guidance values have been set by the Joint Food and Agriculture Organization of the United Nations/World Health Organization (FAO/WHO) Expert committee on Food Additives (JECFA) [5] (and by European Food Safety Authority (EFSA) [6].

Considering the growing worldwide demand for food and the climatic and environmental changes [3], it is necessary to develop rapid methods for the large-scale monitoring and assessment of food safety. There are different methods for human exposure assessment, which may be based on the analysis of contamination levels in food and dietary surveys (indirect methods) [7,8] or direct human biomonitoring studies (HBM), including the analysis of food contaminants in human urine or blood. However, both types of studies are very time-consuming, expensive, and require the recruitment of numerous respondents to participate in the studies. Diet survey studies are associated with participant bias and inaccurate assumptions, such as subjective views of food quantities ingested or cooking practices applied. In addition, there are difficulties associated with the extrapolation of individual results from those studies to the targeted population, since the analysis of some matrices may provide only limited understanding regarding the exposure to a substance, related only to the specific time of collection.

Wastewater-based epidemiology (WBE) is a promising biomonitoring approach with the potential to provide direct information on the human intake and exposure to food-borne and environmental chemicals. It is based on the analysis of specific human metabolites as biomarkers in urban wastewater (WW). The content of biomarker in WW corresponds to the excreted biomarker content in urine. There have been many studies that confirmed the correlation of biomarker content in urine with the daily intake [8,9,10]. The main challenges of these applications are related to the selection of a suitable biomarker, lack of information about the kinetics of absorption and excretion of mycotoxins in humans, and the general uncertainty regarding daily intakes estimation. It should be mentioned as well that this approach is applicable only for compounds that are water-soluble and excreted through urine [11,12]. Despite the many challenges, this approach was shown to be comparable with other studies [11].

There have been several studies related to mycotoxin contamination of WWs. Most of them were aimed at the determination of mycotoxin content in different water and WW types [11,13,14,15,16,17,18], but only a single study used the data obtained for assessing human exposure to mycotoxins by measuring specific biomarkers [11]. Within the study by Gracia-Lor et al. (2020) [11], the concentration of mycotoxin biomarkers in raw WWs was determined and the daily intake of mycotoxins per person was calculated, taking into account the daily flow rate of WW and the number of inhabitants. In our study, we propose to augment the WBE approach with a normalization method using a population biomarker, 5-hydroxyindoleacetic acid (5-HIAA). This type of normalization method has been used previously for detecting certain other compounds and adjusted for different population biomarkers [12]. Subsequently, the elaborated model was applied to the assessment of deoxynivalenol (DON) intake, and for the provision of preliminarily data about the exposure of Latvian population to enniatins.

Mycotoxin-producing *Fusarium* species are major pathogens in cereals like wheat, oats, barley, and maize. Deoxynivalenol is one of the most frequently detected mycotoxins in wheat [19]. The toxicity of DON has been widely studied and both JECFA and EFSA have derived a tolerable daily intake (TDI) of 1µg/kg b.w. per day and range for chronic dietary exposure (214–1014 ng/kg b.w. day) [5,6]. 

ENNs are commonly found in several grains and their derived products. Although the cytotoxicity of enniatin toxins has been assessed in vitro, separate studies in vivo have not confirmed the adverse effects. Among the four enniatins (ENNA, ENNA1, ENNB, ENNB1), ENN B is currently the most studied since it has been the most-often detected in unprocessed and processed grains from European countries. Concentrations of ENN B in grains range from a few μg/kg to over mg/kg. The EFSA Panel on Contaminants in the Food Chain concluded that acute exposure to beauvericin and enniatins does not present any concern for human health. At the same time, there may be risks related to chronic exposure to these mycotoxins [2].

## 2. Results and Discussion

### 2.1. Optimization of the Sample Preparation and Extraction Procedure

Three volumes, namely 333, 666, and 1000 mL, were tested to determine the optimal sample volume for the solid phase extraction (SPE) procedure. The obtained results showed that the signal became noticeably stronger upon increasing the sample volume from 333 to 666 mL, but then the signal remained unchanged when the sample volume was further increased from 666 to 1000 mL. This may be related to the richness of effluent matrix and the limited capacity of the SPE (see Supplementary Figures S3 and S4). From the obtained results, it was confirmed that the sample volume of 666 mL of sample is optimal, and for simplicity, 500 mL of sample was further used.

We observed that lowering the pH value of the sample improved the recovery for many analytes [11,16,20]. Therefore, formic acid was added to pH 4 for all samples before filtration.

One of the most important stages of the method development was the selection of effective and suitable SPE protocol. Most of the previously published methods for mycotoxin extraction from water matrices have used Oasis HLB columns [11,16,17]. In our study, Oasis HLB (Waters, Milford, MA, USA) columns, Strata-X (Phenomenex, Torrance, CA, USA), and Strata C18-E (Phenomenex, USA) SPE cartridges were tested. Oasis HLB and Strata-X are polymeric reversed-phase sorbent cartridges that can be used for the extraction of acidic, basic, and neutral compounds. Strata C18-E is a silica-based end-capped C18 sorbent that can be used for extraction of hydrophobic or polar organic analytes from aqueous matrices.

The obtained experimental results confirmed the high absolute recovery values for Oasis HLB (80%) and Strata-X (104%) for all selected mycotoxins. Although the median extraction with Strata C18-E column was 69% for mycotoxins of the BEA and ENN group, unacceptably low recovery was obtained for DON, only 9% (see Supplementary Table S1 and Figure S2).

During the initial linearity measurement tests, it was observed that BEA recovery from the SPE was insufficient at higher concentrations, so the elution volume was increased from 5 mL to 10 mL. 

Various experiments were performed to find the optimal reconstruction solution. Poor absolute recoveries were observed for non-polar compounds using a high-water content reconstruction solution, while increasing the organic fraction in the solution improved these values. This effect is particularly significant in the presence of sample matrix. The aim was to select a reconstruction solution that dissolved the dry extract well, while the final composition did not impair the peak shape on the column and the compounds were stable in the selected reconstruction solution.

As a result, 17% (*v/v*) methanol solution in water with 1% acetic acid and 250 mM ammonium acetate was selected for the reconstitution in the analysis of DON, and 50% (*v/v*) methanol solution in water with 1% acetic acid and 250 mM ammonium acetate was selected for the BEA and ENN group. The reconstruction workflow is described in Section 2.3.

### 2.2. Chromatographic Separation and MS Detection

The chromatographic separation of the mycotoxins was optimized with respect to the column type, mobile phase, and gradient program to achieve the greatest possible selectivity and sensitivity. 

Ten analytical columns (Phenomenex and ThermoFischer (Waltham, MA, USA)) with different type of sorbents (C18, phenyl, C12, pentafluorophenyl, phenylhexyl, and graphite carbon) were compared by the sorption coefficient and the number of theoretical plates. Tests were performed in isocratic water-acetonitrile mode with a flow rate of 1 mL/min. From the obtained signal width, retention times and used column dimensions, the sorption coefficient and the number of theoretical plates were calculated. The determination of column efficiency defined our choice of analytical column. A porous graphitic carbon Hypercarb™ column was selected as the stationary phase for the separation of DON because of the high number of theoretical plates, well-resolved peak shape, sensitivity, and signal intensity. The Kinetex PFP column was chosen for the chromatographic separation of beauvericin and enniatins due to the well-resolve peak shape and signal intensity that were obtained with this column.

The mycotoxins were ionized to either their [M−H]^−^, [M−H]^+^, [M+Ac]^−^, [M+NH_4_]^+^, or [M+Na]^+^ ions. The polarity showing the more abundant precursor ions was selected for each analyte. DON and U-[^13^C_15_]-deoxynivalenol IS-DON were measured in the negative ionization mode, while other mycotoxins were measured in the positive ionization mode (see Supplementary Figure S1).

### 2.3. Validation of the Optimized Method

#### 2.3.1. Linearity

Linearity was initially tested using standard solutions with the appropriate phase composition (described in Section 4. Materials and Methods) and the correlation factor of R^2^ > 0.99 was observed. During the initial experiments, poor linearity of the method was obtained for BEA (R^2^ < 0.80). However, raising the volume of eluting solution from 5 to 10 mL improved the linearity to R^2^ = 0.99 (see the Supplementary Table S5).

#### 2.3.2. Evaluation of Matrix Effects

According to other studies [16,17], the most common characteristic of wastewater matrix is the ion suppression. In order to assess this phenomenon in our study, three different WW samples were selected and prepared as described above. Ion suppression was quantified as reaching up to 96%. Such high interference might be explained by high output of organic material from wastewater. On average, the strongest matrix effects were observed for BEA (78–96%) and the lowest for ENNA (24–51%) (see Supplementary Table S2). These results were consistent with other studies [16,17]. Because of high matrix effects, mycotoxins were quantified by standard addition procedure, not external calibration, thus eliminating the effect of matrix on the accuracy of the result.

#### 2.3.3. Absolute and Relative Method Recoveries

Absolute method recoveries ranged from 54% to 138% at 5 ng/L for BEA and ENN group mycotoxins. The range of absolute recoveries was more extreme at the lowest concentration level, from 27% to 134%. The results at the concentration levels of 5 ng/L and 10 ng/L were obtained in two rounds of experiments and demonstrated the repeatability of method (see Supplementary Tables S3 and S4). At the lower concentration level of 5 ng/L, the absolute recoveries for DON were in the range of 84–122% (median at 93%), and at 50 ng/L and 200 ng/L both were in the range of 99–118%, with the median values of 104% and 115%, respectively. 

For the analysis of DON, an isotope labeled internal standard (ILIS) was used, compensating for the differences in preparation process and during ionization. The obtained relative method recoveries for DON were from 60% to 120% (the median value of 102%) at the 5 ng/L concentration level. At higher concentration levels, the relative recoveries were in the range from 72–116% (median values in the range of 90–113%). These results were in a good agreement with an earlier study [16]. On the basis of the obtained results, it was decided to apply the ILIS-corrected quantification procedure for DON and the standard addition method for other compounds.

#### 2.3.4. LOD, LOQ and Precision

In this study, satisfactory limit of quantification (LOQ) values were obtained for mycotoxins of the BEA and ENN group, ranging from 0.13 to 0.47 ng/L and for DON—6.4 ng/L. In similar studies, the limit of detection (LOD) of 3.4 ng/L was obtained for BEA and 1.2 ng/L for DON [16]. The method proposed in our study indicated the lowest LOD level for the determination of BEA in WW, as well as for the first time offered a validated determination of ENN mycotoxins in WW. The overall method precision ranged from 1.3 to 7% (see Supplementary Table S5). These results were in good agreement with a similar study [16].

### 2.4. Occurrence of Mycotoxins in Wastewater

During the six-week monitoring, six compounds were monitored in raw WW samples (see Table 1). Mycotoxins of the DON and ENN groups were detected in numerous samples. However, the presence of BEA was detected less frequently and at lower concentrations than expected from previous studies [13,16]. Although fumonisins were often included in the analyte scope of other methods, these compounds were not included in our method since they were not found in the samples during a preliminary study. This may be explained with climate conditions in Latvia, since fumonisin-producing moulds are more common in the warm climate of the Southern Europe [21]. 

The detected concentrations of DON in WW samples in our study were compatible with the data from other authors (see Supplementary Tables S8 and S10). In two studies from Switzerland, 14 samples were tested from two different regions and DON was detected with 100% frequency in the range of 37–122 ng/L [17]. In another study using data from 411 WW samples, DON was detected in 54% of samples at the average concentration of 75 ng/L [18]. There are no data on the distribution of enniatins in WW, but enniatins have been frequently found in urine samples. Enniatin B has been detected in urine at 0.006 to 0.391 ng/mL concentrations [22] and enniatin B1 was detected at 0.007 to 0.429 ng/mL [23]. It should be considered that these studies were from Italy where the occurrence of enniatin may be different than in the northern Europe. Considering the total dilution factor in WW (100–400 times) [11], the concentrations obtained during our study largely agree with the results from the aforementioned urine analysis.

### 2.5. Wastewater Based Epidemiology for Tracking Human Exposure to Mycotoxins

Previously, 5-Hydroxyindoleacetic acid (5-HIAA) is a suitable biomarker for assessing population size and its application has been recommended by several authors [24,25]. Moreover, 5-HIAA is the main serotonin metabolite among human endogenous chemicals. Its excretion rate depends on the age and sex: young children excrete approximately 3 mg/day, adults 5–10 mg/day, and the elderly 7–10 mg/day. Women have a slightly lower rate of 5-HIAA excretion [26,27,28]. It has been calculated based on data from 17 WWTPs [25] that the excretion rate of 5-HIAA (ER_5-HIAA_) is 4.16 ± 0.56 mg/day/person and this value was used in our subsequent calculations. In this study the prevalence of 5-HIAA in wastewater samples was 100% frequent and concentrations ranged from 8.68 to 19.5 µg/L with 14.8 µg/L and 14.6 µg/L median and average concentrations, respectively (see Supplementary Table S10). LOD and LOQ values of the 5-HIAA analysis were 0.3 µg/L and 1 µg/L, respectively. 

A specific excretion factor (CF) definition is offered in previous studies [29,30]. In a study of deoxynivalenol and deoxynivalenol-3-glucoside metabolization and excretion processes in humans [31], it was suggested to use a correction factor of 1.6 (range 1.5–1.7) as cumulated excretion factor for exposure of DON calculations. It was estimated based on the cumulative excretion of total DON after an exposure to a mixture of DON and deoxynivalenol-3-glucoside. In a study [11] about the excretion rates of DON based on three different sources, a correction factor of 1.44 was calculated. Considering the earlier studies, our selected average value for the correction factor (${\mathrm{CF}}_{\mathrm{DON}})$ was 1.5. 

The daily intake of DON per person was calculated (Equation (1)) from the observed concentration of DON in WW samples (ng/L), the responding content of 5-HIAA in sample (ng/L), CF_DON_, and the average excretion rate of 5-HIAA (ER_5-HIAA_) (mg/day per person).

DON_intake_ = Conc._DON_·ER_5-HIAA_·CF_DON_/Conc._5-HIAA_ (1)

**Figure 1:**
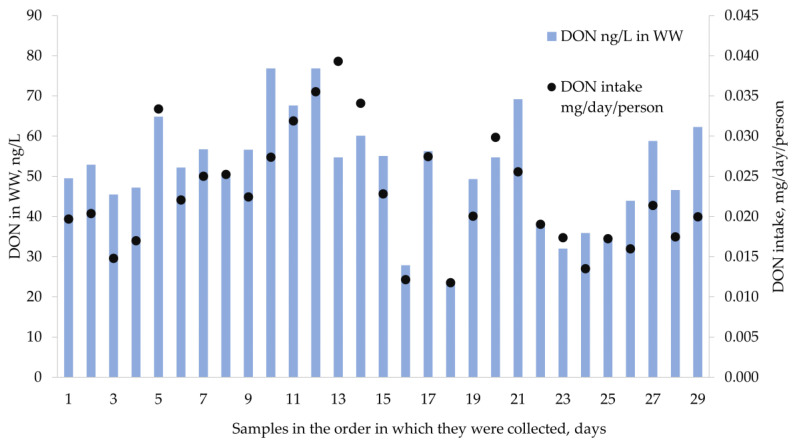
The detected concentrations of DON (ng/L) in wastewater and normalized concentration of DON (mg/day/person) over 6 weeks.

The average daily intake of DON was estimated from 29 samples at 0.023 mg/day per person. The data were in the range from 0.012 mg/day/person to 0.039 mg/day/person and the median value was 0.021 mg/day per person (see Figure 1). 

Several conditions may affect the homogeneity of the obtained data. For example, the stability of mycotoxins in a sewage system might be influenced by temperature and wastewater flow changes and other factors [32]. Although the stability of mycotoxins in the sewage system is a very critical aspect, it has not been studied before and future studies are needed to develop for this type of exposure studies. 

In addition, the results are also influenced by analytical deviations and differences in exposure to mycotoxins of the Riga population considering the variability in batches of food consumed. However, the observed variability is typical or even lower in comparison with uncertainties of the risk assessment studies based on food consumption data or human biomonitoring.

**Figure 2:**
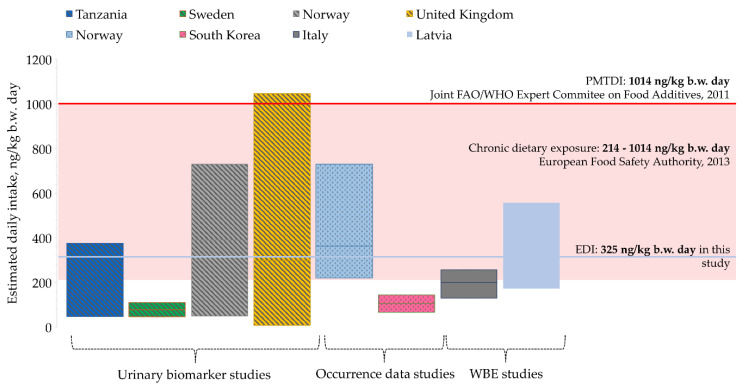
Comparison of the estimated daily intake of DON with other studies and with the reported health-based guidance values.

The reliability of the data was assessed by comparing the WBE results with other studies that were focused on estimating the daily intake of DON (see Figure 2). Assuming an adult body weight of 70 kg [33], the average estimated daily dose of DON was 325 ng/kg b.w.

The daily intake of DON in Tanzania was estimated at 47–376 ng/kg b.w. day based on urinary biomarker studies of children [34]. Similarly, the average daily intake of DON for adolescents in Sweden was estimated at the level of 46–110 ng/kg b.w. day [9]. These values were lower than those obtained in Norway and United Kingdom (390 ng/kg b.w. day and 298 ng/kg b.w. day, respectively) [8,10]. Exposure estimation based on food consumption showed generally higher data than DON exposure values calculated from urinary DON concentrations [8]. In South Korea, the intake of DON was assessed at the rather low level of 66–144 ng/kg b.w. day [7]. An average value of 200 ng/kg b.w. day was obtained in a WBE study in Italy [11]. Climate plays an important role in the occurrence of mycotoxins, so the results obtained in our study should be expected to be similar to data from the UK and Norway [21] (see Supplementary Table S9). 

Although the provisional maximum tolerable daily intake (1014 ng/kg b.w. day) set by the Joint FAO/WHO Expert committee on Food Additives was not exceeded [5], the average estimated daily intake was in the range established for chronic dietary exposure set by the European Food Safety Authority, 2014 (214–1014 ng/kg b.w. day) [6]. A visual representation of the data is displayed in Figure 2. 

Very little information is currently available about the kinetics of absorption and excretion of enniatins and beauvericin in any kind of species. It has been reported that the absorption of enniatin B in pigs after intra-gastric dosing is 91% [2]. Mycotoxins of the ENN group have been frequently detected in human blood serum and urine. Thus, in two studies from Italy [22,23], enniatin B1 was observed of 84% and 94% of urine samples in the range of 0.006–0.391 ng/mL and 0.007–0.429 ng/mL, respectively. Enniatin B was frequently detected in urine samples [35], with the average concentration of 0.017 ng/mL. Although there is currently no information on the rate of urinary excretion of enniatins, we can assume that it might be in the range of 5–50%. By applying this assumption (see Supplementary Table S11), we summarized the daily intake values for ENN group mycotoxins in Table 2.

The EFSA Panel on Contaminants in the Food Chain concluded that acute exposure to beauvericin and enniatins does not present any concern for human health. At the same time, there may be risks related to chronic exposure to these mycotoxins. The chronic dietary exposure to the sum of enniatins across the European countries has been estimated to be in the range of 0.91 (LB minimum 95th percentile)—3.28 (UB maximum 95th percentile) μg/kg b.w. day for adults [2]. In the worst-case scenario (if excretion factors are 5% or lower), the sum of average provisional daily intake of enniatins is 1.35 μg/kg b.w. day, indicating a potential risk of chronical exposure. 

## 3. Conclusions

A sensitive analytical methodology based on solid phase extraction and HPLC-MS/MS was elaborated in this study for the analysis of DON, enniatins, and beauvericin in wastewater (WW) samples. For the first time, a validated method for enniatin group mycotoxin determination in WW was developed. The lowest LOD for beauvericin in WW was achieved. A total of 29 samples taken for six weeks from June to early August from influent at a sewage treatment plant in Riga, Latvia indicated the average concentration of DON at the level of 52 ± 9 ng/L. The concentrations of enniatins were in the range of 0.02–27.7 ng/L. For the first time, the population exposure to mycotoxins was assessed based on the normalization of mycotoxin content in WW to the content of 5-HIAA excretion. The calculated intake of DON by the population of Riga was estimated at 325 ng/kg b.w. day and was in line with the data obtained in human biomonitoring and diet surveys in other countries. Our study represents a faster and more economically reasonable approach in mycotoxin risk assessment studies. 

## 4. Materials and Methods

### 4.1. Reagents and Analytical Standards

Formic acid (≥99%) and acetic acid (≥99.8%) were obtained from VWR Chemicals (Radnor, PA, USA). Methanol (MeOH, LC-grade) and 2-propanol (iPr, LC-grade) were purchased from Merck (Darmstadt, Germany). Ammonium acetate (≥99%) was supplied by Ing. Petr Švec—PENTA (Prague, Czech Republic). Tetrahydrofuran (THF, ≥99%) was purchased from Sigma-Aldrich (St. Louis, MO, USA). Dimethylformamide (DMFA, ≥99.8%) was purchased from Supelco. Deionized water was further purified with a Milli-Q-gradient academic A10 water purification system from Millipore ((Darmstadt, Germany). 

Mycotoxin standards, including U-[^13^C_15_]-deoxynivalenol (IS-DON) (≥99.5%) and deoxynivalenol (≥98.3%), were purchased from Biopure (Guntramsdorf, Austria). Beauvericin (>95%), enniatin A (>99%), enniatin A1 (>99%), enniatin B (>99%), and enniatin B1 (>99%) were supplied by Cayman Chemical (Ann Arbor, MI, USA). 

Stock standard solutions were prepared by dissolving crystalline standard of DON in acetonitrile, while beauvericin, enniatin A, enniatin A1, enniatin B, and enniatin B1 were diluted in DMF. The individual stock solutions had concentrations ranging from 250 to 1100 ng/μL.

Multi-component stock solutions were prepared in acetonitrile at the concentration of 0.125 ng/μL. The internal standard solution was diluted with 1:1 (*v/v*) mixture of acetonitrile and Mili-Q water in order to obtain a concentration of 0.500 ng/μL. All mixtures of compounds were stored at −20 °C in darkness.

### 4.2. Wastewater Sampling

WW samples were collected from the inlet of one WW treatment plant serving Riga, the capital city of Latvia. The sample collection was performed every working day for 6 weeks from the early July to August, 2021, covering a population of 697,000 inhabitants. The samples were immediately frozen, stored in the dark, and analyzed within 2 weeks. The data from the stability test indicates RSD for the content of analytes of up to 47%, but no degradation trend was observed (see Supplementary Table S7). Sample preparation and instrumental analysis procedures used for the 5-HIAA determination in our study were described previously by Pugajeva et al. [36].

### 4.3. Sample Preparation

Before filtration, the pH was adjusted to between 4.5 and 5.5 by adding ≥ 85% formic acid. Exactly 500 mL of WW sample was spiked with 10 µL of the isotope-labelled internal standards (ILIS) mixture and another 500 mL aliquot in the case of calibration samples was spiked with the same volume of ILIS and external standard mixture. The samples were shaken vigorously and then filtered (glass microfibre filters, 47 mm, Filtres Fioroni SA, France) by vacuum filtration.

### 4.4. Solid Phase Extraction

The filtered and spiked samples (500 mL) were concentrated and purified by solid phase extraction procedure (SPE) using Strata™-X 33 µm Polymeric Reversed Phase cartridges, 6 mL, 500 mg (Phenomenex, Torrance, CA, USA). The SPE cartridges were sequentially conditioned with 6 mL of Milli-Q water, 6 mL of MeOH, and 6 mL of Milli-Q water. The WW samples (500 mL) were passed through the SPE system at the average flow rate of 5 mL/min. Without washing step, the cartridges were dried for 30 min and the analytes were eluted with 10 mL of 1% formic acid in methanol. The aliquots were collected in 15 mL polypropylene test tubes (Sarstedt, Nümbrecht, Germany) and were evaporated to dryness under gentle nitrogen stream at 60 °C. The extracts were reconstituted in 120 µL of MeOH/Milli-Q water (50/50, *v/v*) with 250 mM ammonium acetate and 1% acetic acid, well vortexed and shaken. After centrifugation, a 20 µL aliquot was transferred to a glass vial and an additional 30 µL of reconstitution solution was added. After thorough mixing, the solution was filtered through centrifugal filters (PVDF, pore size 0.22 µm, Durapore, Merck Millipore Ltd., St. Louis, MO, USA), transferred to a new glass vial, and used for the analysis of BEA and ENN group mycotoxins. For the analysis of DON, 100 µL of extract was left in sample test tube after the first reconstitution. In the same test tube, 200 µL of Milli-Q water with 250 mM ammonium acetate and 1% acetic acid were added. After thorough shaking and centrifugation, the extract was filtered and transferred to a glass vial. 

### 4.5. LC-MS/MS Analysis

Analyses were performed on a TSQ Quantiva MS/MS coupled to Thermo Scietific Ultimate 3000 HPLC instrument (Thermo Scientific, Waltham, MA, USA). Two different chromatographic runs were used for the separation and quantification of mycotoxins. 

#### 4.5.1. Analysis of Deoxynivalenol

Chromatographic separation was carried out using a Hypercarb (2.1 mm × 100 mm, 5.0 μm) column at 60 °C, using an injection volume of 100 μL. The mobile phase consisted of a 5 mM ammonium acetate solution containing 0.2% acetic acid in Milli-Q water (eluent A), 5 mM ammonium acetate solution containing 0.2% acetic acid in MeOH (eluent B), and THF (eluent C). A flow rate of 0.50 mL/min was used. The following gradient conditions were applied: column equilibration 100% eluent A in 15 min; 0.00 min, 0% B (100% A, 0% C); 40.00 min, 30% B (70% A, 0% C); 40.10 min, 50% B (50% A, 0% C); 42.50 min, 50% B (50% A, 0% C); 42.50 min, 0% B (0% A, 100% C); 52.95 min, 0% B (0% A, 100% C); 53.00 min, 100% B; 58.00 min, 100% B; 65 min, 50% B (50% A, 0% C).

LC-MS interface conditions for the ionization of DON in the negative and positive ESI mode were as follows: needle voltage from −2500 to +4000 V; sheath gas 35 Arb; aux gas 8.5 Arb; sweep gas 0.5 Arb; ion transfer tube temperature 325 °C; vaporizer temperature 300 °C. The main fragments were identified using the selected reaction monitoring (SRM) (see Supplementary Table S6). The properties of SRM were as follows: dwell time 222 ms; Q1 and Q3 resolutions 1.2 FWHM; CID gas 1.5 mTorr; chromatographic peak width 30 s.

#### 4.5.2. Analysis of the Mycotoxin Group including Beauvericin and Enniatins

Chromatographic separation was carried out on a Kinetex PFP (3.0 mm × 100 mm, 1.7 μm) column at 40° C, using an injection volume of 25 μL. The mobile phase consisted of a 5 mM ammonium acetate solution containing 0.2% acetic acid in Milli-Q water (eluent A), 5 mM ammonium acetate solution containing 0.2% acetic acid in MeOH (eluent B), isopropanol (eluent C), and MeOH:H_2_O (1:1, *v/v*, eluent D). The flow rate was in the range from 0.25 to 0.80 mL/min and the following gradient conditions were applied: column equilibration: −6.250 min, 0.80 mL/min 100% eluent A; −0.010 min, 0.80 mL/min 100% A; −0.009 min, 0.25 mL/min 100% A; 0.00 min, 0.25 mL/min 0% B (100% A, 0% C, 0% D); 15.00 min, 99% B (1% A, 0% C, 0% D); 20.00 min, 99% B (1% A, 0% C, 0% D); 24.00 min, 99% B (1% A, 0% C, 0% D); 24.15 min, 99% B (1% A, 0% C, 0% D); 25.00 min, 99% B (1% A, 0% C, 0% D); 25.01 min, 5% B (0% A, 10 C%, 85% D); 29.99 min, 5% B (0% A, 10 C%, 85% D); 30.00 min, 50% B (50% A, 0% C, 0% D); 32.50 min, 99% B (1% A, 0% C, 0% D); 35.00 min, 99% B (1% A, 0% C, 0% D).

The LC-MS interface conditions for the ionization of beauvericin and enniatin group in the positive ESI mode were as follows: needle voltage, +4000 V; sheath gas, 35 Arb; aux gas, 8.5 Arb; sweep gas, 0.5 Arb; ion transfer tube temperature, 325 °C; vaporizer temperature, 300 °C. The main fragments were identified using SRM (see Supplementary Table S6), and the properties were as follows: cycle time, 0.8 s; Q1 and Q3 resolutions, 1.2 FWHM; CID gas, 1.5 mTorr; chromatographic peak width, 6 s.

### 4.6. Method Validation Parameters

#### 4.6.1. Linearity

The linearity of the method was tested both on standard solutions and with different WW samples (*n* = 3). The calibration range of 2.5–200 ng/L for DON was selected based on the occurrence data published from other studies [17,18]. For other mycotoxins, a narrow calibration range of 0.5–25 ng/L was chosen because low concentrations were expected to be found in WW samples. The linearity of the MS/MS detector was tested with MeOH/Milli-Q water (50/50, *v/v*) with 250 mM ammonium acetate and 1% acetic acid containing mycotoxins at concentrations corresponding to the selected concentration ranges.

#### 4.6.2. Matrix Effect

To evaluate matrix effects (ME), standard solutions were prepared in MeOH/Milli-Q water (50/50, *v/v*) with 250 mM ammonium acetate and 1% acetic acid and three visually different WW samples were selected and prepared (the procedure is described in the sample preparation section) without the addition of standard. After the SPE procedure, mycotoxin standards (and ILIS) were added at the dilution step for both instrumental methods so that the solvent ratio in the injection solution remained constant. For both samples and standard solutions, the selected concentrations were equal. The following concentrations were added: 0.5, 3.56, 6.63, 12.75, 18.88, and 25.00 ng/L for BEA and ENN group analytes and 2.5, 10.0, 25.0, 50.0, 100, and 200 ng/L for DON. Calibration curves were obtained by plotting the measured analyte peak areas against the respective concentration levels in solvent and in the sample matrices. The percentage of ion suppression or enhancement was calculated as follow:

ME = (Slope_matrix_/Slope_solvent−_1)·100% (2)

A negative sign expresses ion suppression, but positive, enhancement. 

#### 4.6.3. Absolute SPE Recoveries 

In order to determine the efficiency of the SPE columns with respect to the extraction of target compounds, a comparison of three columns was performed. Milli-Q water samples (three replicates of 500 mL) were spiked at the concentration levels of 5 ng/L for BEA and ENN group mycotoxins and 25 ng/L for DON. The ILIS for DON (10 µL) was added directly into the 10 mL eluates extracted from cartridges. For the matrix-matched sample, Milli-Q water (500 mL) was passed through each type of SPE columns. The matrix-matched sample eluates were spiked just like other samples. The absolute recovery (AR) of SPE for the analytes was defined as follows:

AR_SPE_ = (Area_Spiked before the extraction_/Area_Spiked after the extraction_)·100% (3)

#### 4.6.4. Absolute and Relative Recoveries of Methods

The procedure for determination of absolute (AR) and relative (RR) method recoveries was described previously by [16] The absolute recoveries were determined for all mycotoxins in various WW samples. Five different samples (visually different and of various origins, at least 2 L each) were collected. Each sample was divided into four fractions of 500 mL. Three fractions were spiked with BEA and ENN group mycotoxins to produce the concentration levels of 0.5, 5.0, and 10 ng/L, and with DON to produce 5.0, 50, and 200 ng/L. One fraction of each sample was tested for native mycotoxin content and contamination. After evaporation at the dilution step, ILIS was added to obtain 10 ng/L concentration. The matrix-matched calibration curve for the determination of method recovery was obtained by preparing an averaged WW sample from five selected samples, which was spiked after the SPE procedure and evaporation to obtain concentration levels equivalent to those of initial samples. The absolute recovery for mycotoxins was calculated as Equations (4) and (5).

AR_without ILIS_ = (Area_Spiked before the extraction_/Area_Matrix-match_)·100% (4)

(5) $${\mathrm{AR}}_{\mathrm{with}\;\mathrm{ILIS}}=\frac{\mathrm{Slopei}.e.\frac{\mathrm{spiked}\;\mathrm{before}\;\mathrm{extraction}}{\mathrm{ILIS}\;\mathrm{added}\;\mathrm{in}\;\mathrm{the}\;\mathrm{end}\;\mathrm{of}\;\mathrm{sample}\;\mathrm{preparation}})}{{\mathrm{Slope}}_{\mathrm{matrix}-\mathrm{match}\;\mathrm{calibration}}}$$

The relative method recovery was determined only for DON, because only for this analyte the ILIS was available. It was calculated as follows:

(6) $$\mathrm{RR}=\frac{\mathrm{Slope}\left(\frac{\mathrm{spiked}\;\mathrm{before}\;\mathrm{extraction}}{\mathrm{ILIS}\;\mathrm{added}\;\mathrm{before}\;\mathrm{extraction}}\right)}{{\mathrm{Slope}}_{\mathrm{matrix}-\mathrm{match}\;\mathrm{calibration}}}$$

#### 4.6.5. Method Precision and Detection Limits

The method precision was obtained by testing one WW sample (spiked at concentration level of 5.0 ng/L for BEA and ENN group and at 50 ng/L for DON) prepared in five times and expressing RSD (%) from the resulting areas of analyte peaks.

The detection limit for the method was determined by testing five different WW samples with added 0.5 ng/L of BEA and ENN group mycotoxins and 10 ng/L of DON. The detection limit was defined as the average of each spiked sample concentration (determined in triplicate) divided by the signal to noise ratio corresponding to the peak at this concentration. For each sample type, 500 mL unfortified matrix was analysed for its native mycotoxin contamination. If natural mycotoxin contamination was detected in test samples, it was taken into account in the LOD and method precision calculations.

## Figures and Tables

**Table 1:** The concentrations of mycotoxins measured in urban WW from Riga, Latvia (n = 29).

	Frequency of Detection (>LOD)	Average, ng/L	Range, ng/L	Median, ng/L	LOD, ng/L	LOQ, ng/L	Precision, %
DON	100%	51.7	23.2–76.9	52.9	1.9	6.4	6
ENNA	90%	3.0 *	LOQ–16.7	2.0 *	0.12	0.40	3
ENNA1	86%	4.2 *	LOQ–27.7	2.5 *	0.14	0.47	4
ENNB	100%	4.6	0.6–9.9	4.1	0.04	0.15	1.3
ENNB1	100%	3.9	1.4–10.1	3.4	0.13	0.43	3
BEA	14%	<LOQ	<LOQ	<LOQ	0.04	0.13	7

* The mean values were calculated using LOQ/2 when the measured values were below LOQ.

**Table 2:** Provisional daily intake of enniatins (µg/kg b.w. day) depending on various excretion factors.

	**ENNA**			**ENNA1**		
Assumed Excretion Factors	5%	25%	50%	5%	25%	50%
Average	0.25	0.050	0.025	0.36	0.072	0.036
Max	1.18	0.24	0.12	1.95	0.39	0.19
Min	0.012	0.0024	0.0013	0.017	0.0034	0.0017
Median	0.15	0.029	0.015	0.21	0.042	0.021
	**ENNB**			**ENNB1**		
Average	0.39	0.079	0.039	0.35	0.069	0.035
Max	0.84	0.17	0.084	1.05	0.21	0.105
Min	0.048	0.0097	0.0049	0.109	0.022	0.0109
Median	0.34	0.067	0.034	0.30	0.060	0.030

## Data Availability

Not applicable.

## Supplementary Materials

The following Supplementary materials can be downloaded at: https://www.mdpi.com/article/10.3390/toxins14020091/s1, Figure S1: LC-MS/MS chromatograms from wastewater samples spiked with 5 ng/L for BEA and ENNs’, 50 ng/L for DON, and 20 ng/L for IS-DON; Table S1: Absolute SPE recoveries of mycotoxins in Milli-Q water standard addition concentration 5ng/L for ENN group and BEA, 25 ng/L for DON; Figure S2: Poor DON recovery is explained by the inability of DON to sorb on the C18 column; Table S2: Ion suppression of mycotoxins in various wastewater extracts; Table S3: Absolute method recoveries and method precision (MP) for DON; Table S4: Absolute method recoveries and method precision (MP) for BEA and enniatins; Table S5: Instrument detection limits (IDL) and method detection limit (MDL) and method limit of quantification (MLOQ); Figure S3: DON peak intensity changes depending on the selected sample volume—333 mL, 666 mL and 1000 mL; Figure S4: BEA and ENN group mycotoxin peak intensity changes depending on the selected sample volume—666 mL and 1000 mL; Table S6: Analytical conditions used in the analysis of the mycotoxins and ILIS; Table S7: Stability of mycotoxins in wastewater calculated as percentage of the concentrations measured at t_0_ (Immediately after the spiking DON 50 ng/L, BEA and ENN group 5 ng/L); Table S8: Mycotoxins found in wastewater; Table S9: DON intake estimates from different studies; Table S10: Mycotoxins found in wastewater; Table S11: Estimated daily intake of DON mg/day per person.
