# Supplementary material for: Application of Wastewater-Based Epidemiology for Tracking Human Exposure to Deoxynivalenol and Enniatins
Source: Toxins (Basel). 2022 Jan 25;14(2):91. doi: 10.3390/toxins14020091 (PMC8878170; doi:10.3390/toxins14020091)
Supplement: Supplementary file 1 [file toxins-14-00091-s001.zip › toxins-1551238-supplementary.pdf]

toxins

MDPI

Supplementary Materials

Application of Wastewater-Based Epidemiology for Tracking Human Exposure to Deoxynivalenol and Enniatins

Zane Berzina, Romans Pavlenko, Martins Jansons, Elena Bartkiene, Romans Neilands, Iveta Pugajeva and Vadims Bartkevics

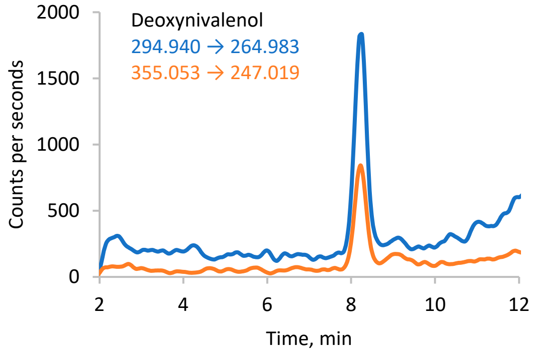

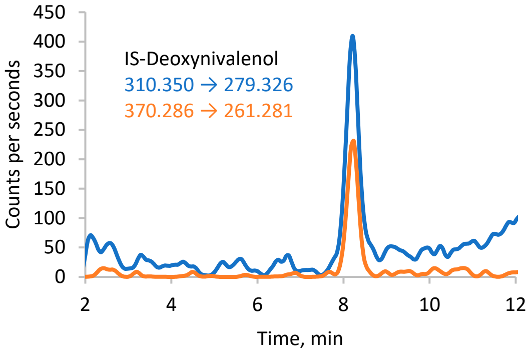

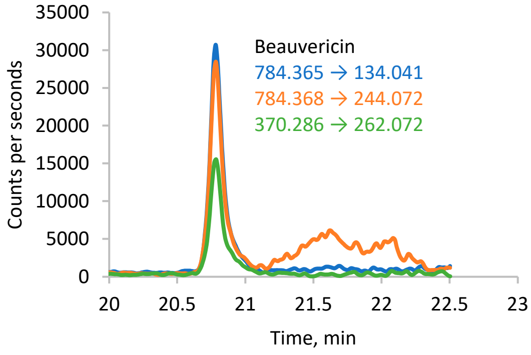

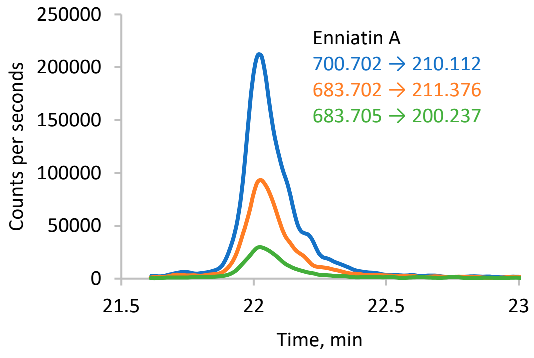

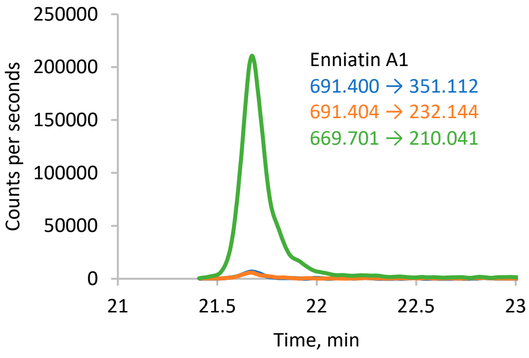

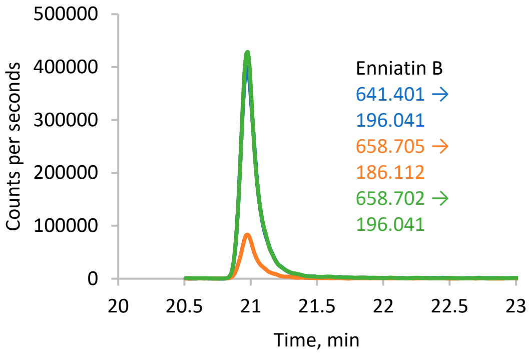

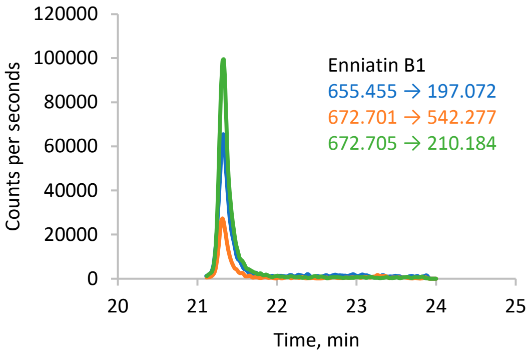

Figure S1. LC-MS/MS chromatograms from wastewater samples spiked with 5 ng/L for BEA and ENNs', 50 ng/L for DON, and 20 ng/L for IS-DON.

Table S1. Absolute SPE recoveries of mycotoxins in Milli-Q water standard addition concentration 5ng/L for ENN group and BEA, 25 ng/L for DON.

	C-18 (%)	STRATA-X (%)	HLB (%)
DON	9	94	94
BEA	59	90	75
ENNA	67	104	92
ENNA1	71	103	82
ENNB	72	106	76
ENNB1	72	106	78
Min. value	9	90	75
Max. value	72	106	94
Median	69	104	80

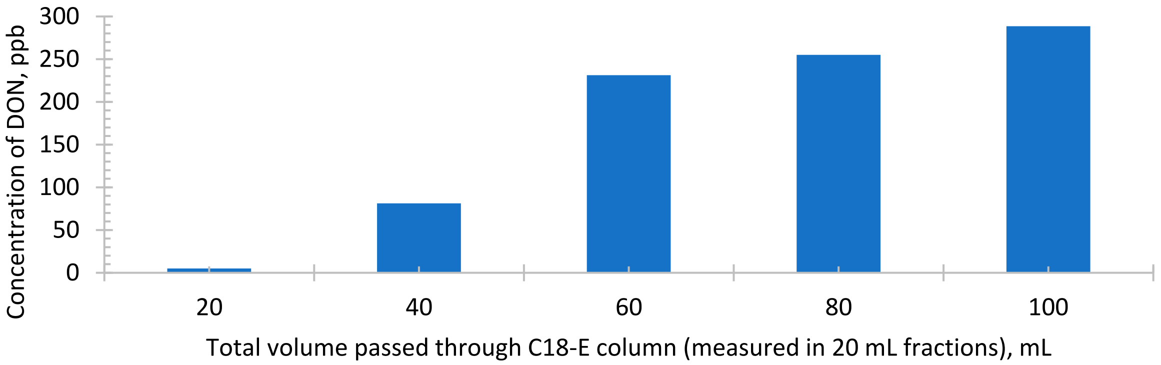

Figure S2. Poor DON recovery is explained by the inability of DON to sorb on the C18 column.

Table S2. Ion suppression of mycotoxins in various wastewater extracts.

	Sample 1 (%)	Sample 2 (%)	Sample 3 (%)	Average (%)	RSD %
DON	8	-	-	-	-
BEA	-96	-78	-91	-88	11
ENNA	-40	-24	-51	-38	35
ENNA1	-75	-51	-56	-61	21
ENNB	-65	-49	-70	-61	18
ENNB1	-67	-38	-64	-56	28

Table S3. Absolute method recoveries and method precision (MP) for DON.

	Concentration (ng/L)	Abs. Method Recov.(%) Min-Max	Abs. Method Recov. (%) (n) Average	Abs. Method Recov.(%) Median	MP (%)	Rel. Method Recov. (%) Min-Max	Rel. Method. Recov. (%) (n) Average	Rel. Method. Recov. (%) Median
DON	5	84-122	97 (5)	93		60-120	109 (5)	102
	25					72-109	90 (4)	90
	50	99 - 118	106 (5)	104	6	92-116	103 (7)	104
	100						96 (1)	96
	200	99 - 118	112 (5)	115		112-113	113 (2)	113

Table S4. Absolute method recoveries and method precision (MP) for BEA and enniatins.

	Concentration (ng/L)	Number of Measurements	Abs. Method Recov. (%) Min-Max	Abs. Method Recov. (%) Average	Abs. Method Recov. (%) Median	MP (%)
BEA	0,5	5	27-64	50	57	
	5	5	94-115	104	101	7
		5	54-96	84	93	
	10	3	71-152	100	78	
		5	43-111	70	66	
ENNA	0,5	5	57-126	75	63	
	5	5	94-103	98	97	3
		5	74-99	89	89	
	10	3	39-169	100	92	
		5	46-109	79	88	
ENNA1	0,5	5	76-120	92	87	
	5	5	94-105	100	98	4
		5	109-128	118	116	
	10	3	45-131	100	124	
		5	50-131	110	126	
ENNB	0,5	5	59-134	93	92	
	5	5	98-102	100	100	1,3
		5	86-127	114	118	
	10	3	69-153	100	78	
		5	56-132	103	114	
ENNB1	0,5	5	70-127	100	103	
	5	5	94-105	100	100	3
		5	100-138	119	116	
	10	3	64-164	100	72	
		5	57-133	105	110	
Min. -Value	0,5/5/10		27/54/39	50	57	1,3
Max. Value	0,5/5/10		134/138/169	119	126	7

Table S5. Instrument detection limits (IDL) and method detection limit (MDL) and method limit of quantification (MLOQ).

	IDL (ng/L)	MDL (ng/L)	MLOQ (ng/L)	Linearity (ng/L)	Correlation Factor (R^2)
DON	0,18	1,9	6,4	2,5-200	0,998
BEA	0,0025	0,039	0,13		0,991
ENNA	0,0102	0,12	0,40		0,987
ENNA1	0,0089	0,14	0,47	0,5-25	0,999
ENNB	0,0055	0,044	0,15		0,991
ENNB1	0,0170	0,13	0,43		0,996
Min. value	0,0025	0,039	0,13		
Max. value	0,170	0,14	0,47		

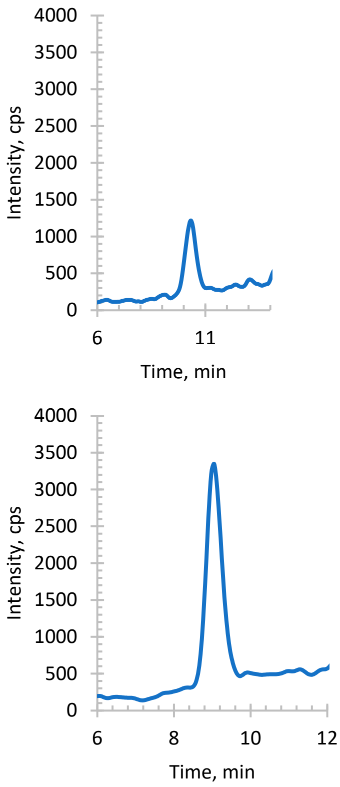

Figure S3. DON peak intensity changes depending on the selected sample volume – 333 mL, 666 mL and 1000 mL.

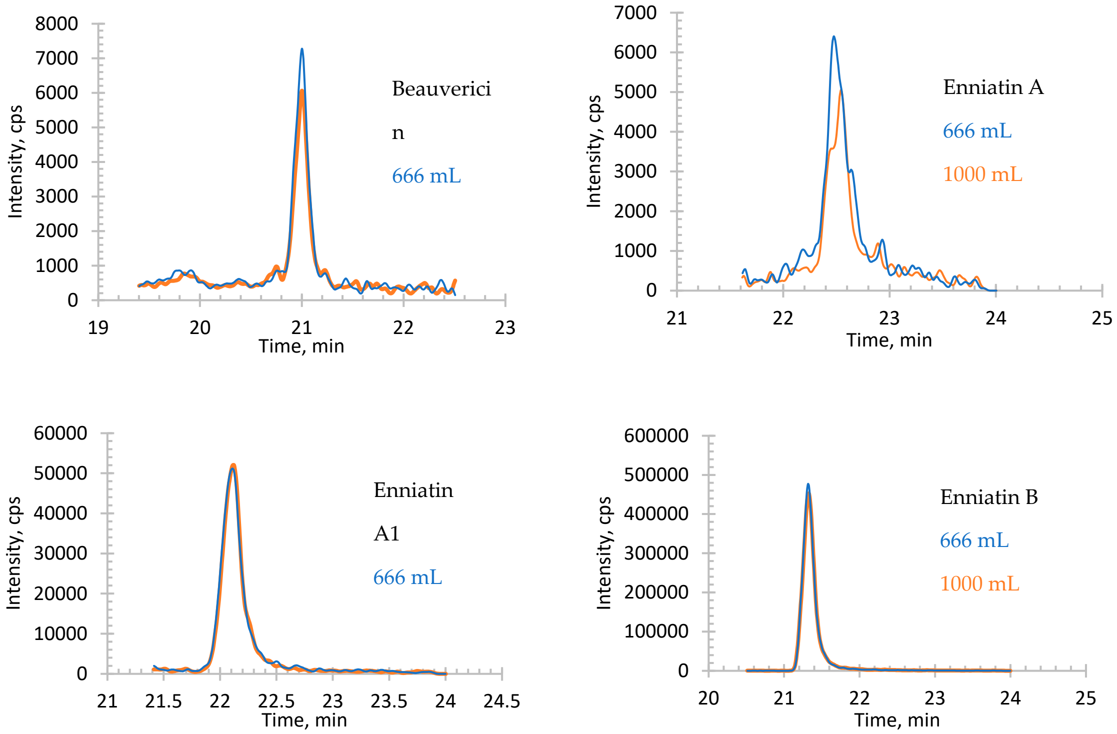

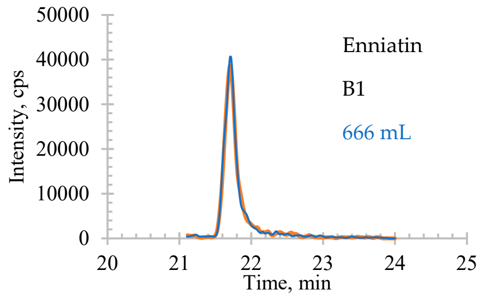

Figure S4. BEA and ENN group mycotoxin peak intensity changes depending on the selected sample volume – 666 mL and 1000 mL.

Table S6. Analytical conditions used in the analysis of the mycotoxins and ILIS.

Compound Name	Retention Time, min	Adduct	Precursor Ion m/z	Product Ion m/z	Collision Energy, eV
Deoxynivalenol	8.20	[M-H]-	294.940	264.982	10.25
		[M+Ac]-	355.053	247.018	14.90
U-[13C15]-Deoxynivalenol	8.21	[M-H]-	310.350	279.325	10.25
Beauvericin	20.8	[M+H]+	784.365	134.040	52.67
		[M+H]+	784.368	244.071	25.83
		[M+NH4]+	801.392	262.071	29.16
Enniatin A	22.0	[M+H]+	683.702	211.375	25.37
		[M+H]+	683.705	200.236	39.48
		[M+NH4]+	700.702	210.111	30.68
Enniatin A1	21.7	[M+H]+	669.701	210.040	24.16
		[M+Na]+	691.40	351.111	51.06
		[M+Na]+	691.404	232.143	55.00
Enniatin B	21.0	[M+H]+	641.401	196.040	24.36
		[M+NH4]+	658.702	196.040	29.82
		[M+NH4]+	658.705	186.111	44.43
Enniatin B1	21.3	[M+H]+	655.455	197.071	23.80
		[M+NH4]+	672.701	542.276	27.60
		[M+NH4]+	672.705	210.183	29.72

Table S7. Stability of mycotoxins in wastewater calculated as percentage of the concentrations measured at t_0 (Immediately after the spiking DON 50 ng/L, BEA and ENN group 5 ng/L).

Compound	Difference after 2 Weeks at 4°C (%)	Difference after 3 Weeks at 4°C (%)	Difference after 2 Weeks at -18°C (%)	Difference after 3 Weeks at -18°C (%)
DON	-2,2	Not Tested	-13	Not tested
BEA	-35	-35	-47	-41
ENNA	-41	-31	-25	-11
ENNA1	-9	+24	-71*	-19
ENNB	+3	+10	-15	+4
ENNB1	+5	0	-15	+24

*Assumed as random error

Table S8. Mycotoxins found in wastewater.

Region	Targeted Compounds	Number of Samples	Positive Samples %	Min – max, ng/L	Mean, ng/L	Method Detection Limit (MDL) ng/L	Reference
Zurich, Switzerland	Beauvericin	4	100	< MDL	-	3.4	[1]
	Deoxynivalenol		100	16.4 – 38.8	26.1	1.2	
	3-acetyl-deoxynivalenol		100	< MDL	-	6	
	Aflatoxin B1,G1,B2,G2,M1		0	Not detected		2.3, 2.1, 6.9, 5.2, 5.1	
	Altenuene, alternariol monomethylether, alternariol, tentoxin					7, 1.7, 1.4, 3.6	
	Ergocornine, ergocryptine					3.9, 2	
	Fumonisin B1, B2+3					6.5, 6	
	Ochratoxin A , B					0.8, 0.7	
	Sterigmatocystin, sulochrin					4, 47.7	
	Citrinine, patulin					0.4, 2.5	
	α, β - zearalenol, Zearalenone					21.5, 31.1, 12.9	
	Diacetoxyscirpenol, HT-2 toxin, neosolaniol, T-2 toxin, verrucarin A					0.5, 4.8, 5.5, 1.2, 10.2	
	3-acetyl-deoxynivalenol 15- acetyl-deoxynivalenol Fusarenone-X Nivalenol					6, n.a.,0.7, 1.6	
Italy	Deoxynivalenol	15 from two different regions	100	-	33.7	10.4	[2]
	Fumonisin B1		100	-	44	2.0	
	Fumonisin B2		100	-	4.6	0.5	
	Fumonisin B3		100	<LOQ-…	3.6	3.3	
	Deepoxydeoxynivalenol (DOM-1), 3-acetyl-deoxynivalenol, 15-acetyl-deoxynivalenol, nivalenol, T-2 toxin		0	Not detected	-	11.6, 20.0, 25.3, 98.0, 1.0,	
	Zearalenone, β - zearalenol					7.6, 8.9	
Spain	Deoxynivalenol	14 from two different regions	100	32-46	39.4	10.4	[2]
	Fumonisin B1		0	Not detected	-	2.0	
	Fumonisin B2		0	Not detected	-	0.5	
	Fumonisin B3		50	-	1.9	3.3	
	Deepoxydeoxynivalenol (DOM-1), 3-acetyl-deoxynivalenol, 15-		0	Not detected	-	11.6, 20.0, 25.3, 98.0, 1.0,	

	acetyl-deoxynivalenol, nivalenol, T-2 toxin						
	Zearalenone, β - zearalenol					7.6, 8.9	
Poland	Zearalenone	Monthly for 13 months	-	-	5.0 (autumn/winter period) 1.0 (spring/summer period)	0.5	[3]
Poland	Zearalenone	Monthly from March to December for 3 years (2010-2012).	100	0.33-19.80	4.1	0.3	[4]
Switzerland	Deoxynivalenol	14 from two different regions	100	37-122	51-113	3.0	[5]
Switzerland	3-acetyl-deoxynivalenol	411	19	<LOQ-367.5	46	5.5	[6]
	Deoxynivalenol		54	<LOQ-1114.5	75	0.8	
	Nivalenol		44	5.0-71.3	13	1.7	
	Beauvericin		36	<LOQ-10.4	2.3	1.4	
	Zearalenone		14	<LOQ-48.4	12.6	6.0	
United States	3-acetyl-deoxynivalenol	3 from three different regions	55	<LOQ – 44.8	18	<6.0	[7]
	Beauvericin		89	<LOQ	-	<3.4	
	Deoxynivalenol		56	< LOQ – 75.4	24.5	<1.3	
	Nivalenol		33	<LOQ	-	(<1.5-<1.6)	
	α - zearalenol		22	<LOQ-817	287.4	(<18.6-<21.5)	
	β - zearalenol		22	<LOQ-1828	301.6	(<4.9-<31.1)	
	Zearalenone		44	<LOQ	-	(<12.3-<12.9)	
	Diacetoxyscirpenol, verrucarin A,		0	-	-	(<0.4-<0.5), (<1.4-<10.2)	

Means were calculated using LOQ/2 when the values were below LOQ, if there wasn't any other information.

Table S9. DON intake estimates from different studies.

Country	Method	Respondents	Average, ng/kg b.w./day	Min, ng/kg b.w./day	Max, ng/kg b.w./day	Reference
Tanzania	Urinary biomarker	166 kids	151	47	376	8
Sweden	Urinary biomarker	1044 pupils	78	46	110	9
Norway	Urinary biomarker	257	390	50	730	10
Norway	Occurrence data during years 2008-2011 of grain-based foods	257	363	220	730	10
Italy	Wastewater based epidemiology	29 samples	200	129	257	2
United Kingdom	Urinary biomarker	35	298	8	1046	11
South Korea	Occurrence data and @Risk program calculations	74 samples	105	66	144	12

Table S10. Mycotoxins found in wastewater.

Sampling date	DON, ng/L	ENNA, ng/L	ENNA1, ng/L	ENNB, ng/L	ENNB1, ng/L	BEA, ng/L	5-HIAA, ng/L
29.06.2021	49.50	0.20	0.235	2.02	1.44	ND	15690
30.06.2021	52.86	2.00	0.235	3.41	2.77	ND	16190
01.07.2021	45.48	0.20	2.10	4.06	2.52	ND	19190
02.07.2021	47.20	2.00	1.35	7.05	3.24	ND	17340
05.07.2021	64.82	0.83	0.235	3.75	5.55	ND	12120
06.07.2021	52.18	1.50	2.50	4.98	2.47	ND	14770
07.07.2021	56.72	1.45	1.47	9.94	3.64	ND	14160
08.07.2021	50.66	1.47	0.235	4.44	3.75	ND	12530
09.07.2021	56.63	0.20	2.86	0.64	4.79	ND	15760
12.07.2021	76.84	0.70	0.62	2.40	1.72	ND	17520
13.07.2021	67.63	1.04	1.34	3.40	2.45	ND	13230
14.07.2021	76.85	1.59	2.74	7.05	4.37	ND	13500
15.07.2021	54.68	2.55	6.69	6.17	6.10	ND	8680
16.07.2021	60.12	2.41	4.08	5.99	9.15	ND	11010
19.07.2021	55.03	2.69	2.28	4.06	3.44	<LOQ	15058
20.07.2021	27.86	3.09	2.52	3.00	2.36	ND	14331
21.07.2021	56.27	3.24	4.87	3.63	2.01	ND	12789
22.07.2021	23.23	7.44	4.32	4.66	3.77	ND	12311
23.07.2021	49.29	4.29	7.12	4.93	4.08	ND	15356
26.07.2021	54.68	5.65	12.4	7.72	10.1	ND	11430
27.07.2021	69.18	16.7	27.7	7.43	5.60	<LOQ	16891
28.07.2021	38.07	6.45	4.85	3.39	3.47	ND	12488
29.07.2021	31.98	3.76	3.96	2.63	2.84	<LOQ	11491
30.07.2021	35.86	6.47	5.93	6.78	5.55	<LOQ	16581
02.08.2021	34.38	1.63	6.29	2.98	3.13	ND	12443
03.08.2021	43.91	2.25	8.56	4.73	4.82	ND	17139
04.08.2021	58.81	1.41	1.64	4.10	3.02	ND	17168
05.08.2021	46.60	1.50	1.58	5.16	3.16	ND	16647
06.08.2021	62.28	1.05	1.21	3.75	2.95	ND	19481

Table S11. Estimated daily intake of DON mg/day per person.

Sampling date	DI of DON, mg/day per person	DI of ENNA, μg/day per person, depending on the provisional excretion factor			DI of ENNA1, μg/day per person, depending on the provisional excretion factor			DI of ENNB, μg/day per person, depending on the provisional excretion factor			DI of ENNB1, μg/day per person, depending on the provisional excretion factor		
		CF 5%	CF 25%	CF50%	CF 5%	CF 25%	CF50%	CF 5%	CF 25%	CF50%	CF 5%	CF 25%	CF50%
29.06.2021	0,020	1,06	0,21	0,11	1,25	0,25	0,12	10,7	2,14	1,07	7,64	1,53	0,76
30.06.2021	0,020	10,3	2,06	1,03	1,21	0,24	0,12	17,5	3,50	1,75	14,2	2,85	1,42
01.07.2021	0,015	0,87	0,17	0,09	9,10	1,82	0,91	17,6	3,52	1,76	10,9	2,19	1,09
02.07.2021	0,017	9,60	1,92	0,96	6,48	1,30	0,65	33,8	6,77	3,38	15,5	3,11	1,55
05.07.2021	0,033	5,70	1,14	0,57	1,61	0,32	0,16	25,7	5,15	2,57	38,1	7,62	3,81
06.07.2021	0,022	8,45	1,69	0,84	14,1	2,82	1,41	28,1	5,61	2,81	13,9	2,78	1,39
07.07.2021	0,025	8,52	1,70	0,85	8,64	1,73	0,86	58,4	11,7	5,84	21,4	4,28	2,14
08.07.2021	0,025	9,76	1,95	0,98	1,56	0,31	0,16	29,5	5,90	2,95	24,9	4,98	2,49
09.07.2021	0,022	1,06	0,21	0,11	15,1	3,02	1,51	3,38	0,68	0,34	25,3	5,06	2,53

12.07.2021	0,027	3,32	0,66	0,33	2,94	0,59	0,29	11,4	2,28	1,14	8,17	1,63	0,82
13.07.2021	0,032	6,54	1,31	0,65	8,43	1,69	0,84	21,4	4,28	2,14	15,4	3,08	1,54
14.07.2021	0,036	9,80	1,96	0,98	16,9	3,38	1,69	43,5	8,69	4,34	26,9	5,39	2,69
15.07.2021	0,039	24,4	4,89	2,44	64,1	12,8	6,41	59,1	11,8	5,91	58,5	11,7	5,85
16.07.2021	0,034	18,2	3,64	1,82	30,8	6,17	3,08	45,3	9,05	4,53	69,1	13,8	6,91
19.07.2021	0,023	14,9	2,97	1,49	12,6	2,52	1,26	22,4	4,48	2,24	19,0	3,80	1,90
20.07.2021	0,012	17,9	3,59	1,80	14,7	2,93	1,46	17,4	3,48	1,74	13,7	2,74	1,37
21.07.2021	0,027	21,1	4,21	2,11	31,7	6,33	3,17	23,6	4,72	2,36	13,1	2,61	1,31
22.07.2021	0,012	50,3	10,1	5,03	29,2	5,83	2,92	31,5	6,30	3,15	25,5	5,09	2,55
23.07.2021	0,020	23,3	4,65	2,33	38,6	7,71	3,86	26,7	5,34	2,67	22,1	4,42	2,21
26.07.2021	0,030	41,1	8,23	4,11	90,6	18,1	9,06	56,2	11,2	5,62	73,6	14,7	7,36
27.07.2021	0,026	82,3	16,5	8,23	136	27,3	13,6	36,6	7,32	3,66	27,6	5,52	2,76
28.07.2021	0,019	42,9	8,60	4,30	32,3	6,46	3,23	22,6	4,52	2,26	23,1	4,62	2,31
29.07.2021	0,017	27,2	5,45	2,72	28,7	5,74	2,87	19,1	3,81	1,91	20,6	4,11	2,06
30.07.2021	0,013	32,5	6,49	3,25	29,8	5,95	2,98	34,0	6,81	3,40	27,9	5,57	2,79
02.08.2021	0,017	10,9	2,18	1,09	42,1	8,41	4,21	19,9	3,99	1,99	20,9	4,19	2,09
03.08.2021	0,016	10,9	2,18	1,09	41,6	8,31	4,16	22,9	4,59	2,30	23,4	4,68	2,34
04.08.2021	0,021	6,83	1,37	0,68	7,95	1,59	0,79	19,9	3,97	1,99	14,6	2,93	1,46
05.08.2021	0,017	7,50	1,50	0,75	7,90	1,58	0,79	25,8	5,16	2,58	15,8	3,16	1,58
06.08.2021	0,020	4,48	0,90	0,45	5,17	1,03	0,52	16,0	3,20	1,60	12,6	2,52	1,26
Average:	**0,023**	**17,6**	**3,53**	**1,76**	**25,2**	**5,04**	**2,52**	**27,6**	**5,52**	**2,76**	**24,2**	**4,85**	**2,43**
Min:	**0,012**	**0,87**	**0,17**	**0,09**	**1,21**	**0,24**	**0,12**	**3,38**	**0,68**	**0,34**	**7,64**	**1,53**	**0,76**
Max:	**0,039**	**82,3**	**16,5**	**8,23**	**136**	**27,3**	**13,6**	**59,1**	**11,8**	**5,91**	**73,6**	**14,7**	**7,36**
Median:	**0,021**	**10,4**	**2,06**	**1,03**	**14,6**	**2,93**	**1,46**	**23,5**	**4,72**	**2,36**	**20,9**	**4,19**	**2,09**

References

(1) Schenzel, J.; Schwarzenbach, R. P.; Bucheli, T. D. Multi-residue screening method to quantify mycotoxins in aqueous environmental samples. *J. Agric. Food Chem.* **2010**, *58*, 11207–11217. DOI: 10.1021/jf102737q.

(2) Gracia-Lor, E.; Zuccato, E.; Hernández, F.; Castiglioni, S. Wastewater-based epidemiology for tracking human exposure to mycotoxins. *J. Hazard. Mater.* **2020**, *382*, 121108. DOI: https://doi.org/10.1016/j.jhazmat.2019.121108.

(3) Gromadzka, K.; Waśkiewicz, A.; Goliński, P.; Świetlik, J. Occurrence of estrogenic mycotoxin – zearalenone in aqueous environmental samples with various nom content. *Water Res.* **2009**, *43*, 1051–1059. DOI: https://doi.org/10.1016/j.watres.2008.11.042.

(4) Gromadzka, K.; Waśkiewicz, A.; Świetlik, J.; Bocianowski, J.; Goliński, P. The role of wastewater treatment in reducing pollution of surface waters with zearalenone / uloga pročišćavanja otpadnih voda u smanjenju onečišćenja površinskih voda zearalenonom. *Arch. Ind. Hyg. Toxicol.* **2015**, *66*, 159–164. DOI: doi:10.1515/aiht-2015-66-2606.

(5) Wettstein, F. E.; Bucheli, T. D. Poor elimination rates in waste water treatment plants lead to continuous emission of deoxynivalenol into the aquatic environment. *Water Res.* **2010**, *44*, 4137–4142. DOI: https://doi.org/10.1016/j.watres.2010.05.038.

(6) Schenzel, J.; Forrer, H. R.; Vogelgsang, S.; Hungerbühler, K.; Bucheli, T. D. Mycotoxins in the environment: i. production and emission from an agricultural test field. *Environ. Sci. Technol.* **2012**, *46*, 13067–13075. DOI: 10.1021/es301557m.

(7) Kolpin, D. W.; Schenzel, J.; Meyer, M. T.; Phillips, P. J.; Hubbard, L. E.; Scott, T.-M.; Bucheli, T. D. Mycotoxins: diffuse and point source contributions of natural contaminants of emerging concern to streams. *Sci. Total Environ.* **2014**, *470–471*, 669–676. DOI: https://doi.org/10.1016/j.scitotenv.2013.09.062.

(8) Srey, C.; Kimanya, M. E.; Routledge, M. N.; Shirima, C. P.; Gong, Y. Y. Deoxynivalenol exposure assessment in young children in tanzania. *Mol. Nutr. Food Res.* **2014**, *58*, 1574–1580. DOI: https://doi.org/10.1002/mnfr.201400012.

(9) Warensjö Lemming, E.; Montano Montes, A.; Schmidt, J.; Cramer, B.; Humpf, H. U.; Moraeus, L.; Olsen, M. Mycotoxins in blood

and urine of swedish adolescents—possible associations to food intake and other background characteristics. *Mycotoxin Res.* **2020**, *36*, 193–206. DOI: 10.1007/s12550-019-00381-9.

(10) Eriksen, G. S.; Knutsen, H. K.; Sandvik, M.; Brantsæter, A.-L. Urinary deoxynivalenol as a biomarker of exposure in different age, life stage and dietary practice population groups. *Environ. Int.* **2021**, *157*, 106804. DOI: https://doi.org/10.1016/j.envint.2021.106804.

(11) Turner, P.; White, K.; Burley, V.; Hopton, R.; Rajendram, A.; Fisher, J.; Cade, J.; Wild, C. A comparison of deoxynivalenol intake and urinary deoxynivalenol in uk adults. *Biomarkers* **2010**, *15*, 553–562. DOI: 10.3109/1354750X.2010.495787.

(12) Ok, H. E.; Kim, H. J.; Cho, T. Y.; Oh, K. S.; Chun, H. S. Determination of deoxynivalenol in cereal-based foods and estimation of dietary exposure. *J. Toxicol. Environ. Health. A* **2009**, *72*, 1424–1430. DOI: 10.1080/15287390903212832.
